# CD28 and TCR differentially impact naïve and memory T cell responses

**DOI:** 10.1093/discim/kyaf006

**Published:** 2025-04-22

**Authors:** Cayman Williams, Dalisay Giovacchini, Alan Kennedy, Neil Halliday, Erin Waters, Maximillian Robinson, Claudia Hinze, David M Sansom

**Affiliations:** UCL Institute of Immunity and Transplantation, Division of Infection and Immunity, London, NW3 2PP, UK; UCL Institute of Immunity and Transplantation, Division of Infection and Immunity, London, NW3 2PP, UK; UCL Institute of Immunity and Transplantation, Division of Infection and Immunity, London, NW3 2PP, UK; UCL Institute of Immunity and Transplantation, Division of Infection and Immunity, London, NW3 2PP, UK; UCL Institute of Immunity and Transplantation, Division of Infection and Immunity, London, NW3 2PP, UK; UCL Institute of Immunity and Transplantation, Division of Infection and Immunity, London, NW3 2PP, UK; UCL Institute of Immunity and Transplantation, Division of Infection and Immunity, London, NW3 2PP, UK; UCL Institute of Immunity and Transplantation, Division of Infection and Immunity, London, NW3 2PP, UK

**Keywords:** co-stimulation, T cell, CD28, T cell memory, T cell receptor

## Abstract

Manipulating CD28 co-stimulation is a key element of anti-tumour immune responses and treating autoimmune diseases. CD28 can reduce the T cell activation threshold but has a complex relationship with T cell receptor (TCR) signalling and an unclear role in specific T cell subsets. Using a series of *in vitro* stimulation assays, we have studied the relative contribution of CD28 and TCR signals in human CD4 + T cell responses. We show that not only the quantity of CD28 co-stimulation but also its intensity relative to TCR differentially impacts the division of naïve and memory T cells. We show that CD28 co-stimulation can have TCR-independent effects on memory T cell phenotype and cytokine production and in some settings can antagonize TCR-driven functions. These data highlight the complex relationship between CD28 co-stimulation and TCR signals and expose clear differences in their use by naïve and memory T cells.

## Graphical Abstract

**Graphical Abstract:**
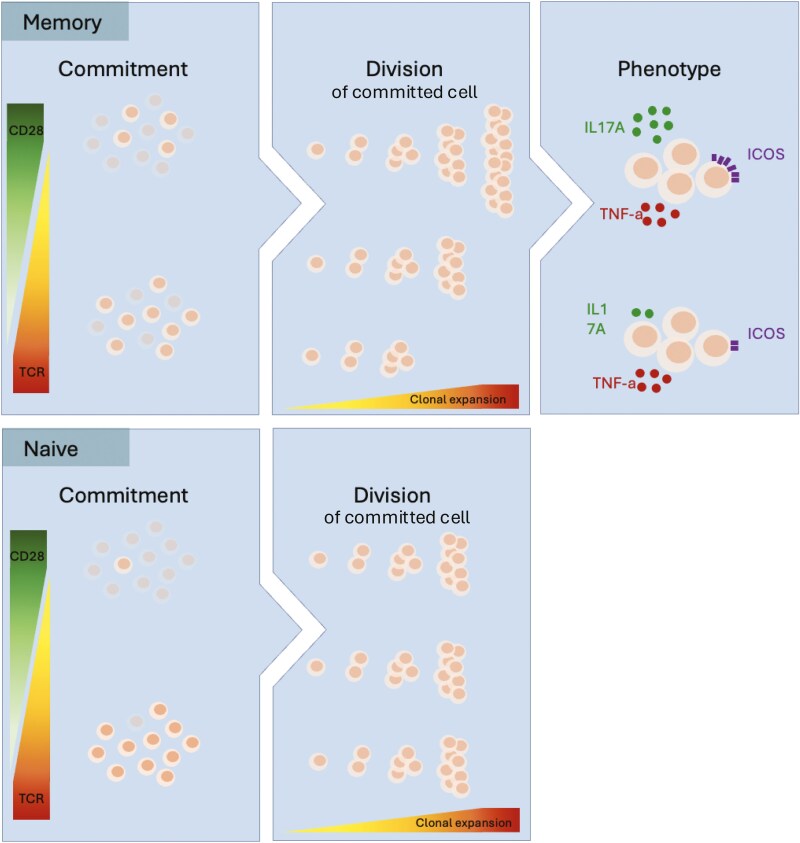

## Introduction

Co-stimulation of T cells via the CD28 receptor is thought to provide important cues for T cell activation. Notably, the quality and quantity of the ligand–receptor interactions vary considerably for both the T cell receptor (TCR) and CD28 receptors. Accordingly, TCR signals vary in relation to the abundance and affinity of peptide–Major Histocompatibility Complex (MHC) interactions, whereas CD28 engagement varies due to affinity and abundance of ligand, which is upregulated by activation of antigen-presenting cells (APCs) and downregulated by CTLA4-dependent removal [1–3]. Previously, studies have suggested that naïve T cells are sensitive to CD28 co-stimulation and require its engagement for their activation [4–11]. In contrast, memory T cells have been suggested to be less dependent on CD28 signals [5, 6, 10–12]. This has given rise to a perception that co-stimulation is more important for naïve T cell responses but less so for memory, although, the evidence for this remains limited. Indeed, the impact of CD28 co-stimulation on memory T cells was spectacularly revealed by the adverse events seen in the CD28 superagonist TGN1412 trial, where effector memory T cells were triggered by CD28 stimulation alone [13–15].

In addition, CD28 co-stimulation is thought to lower the TCR threshold when levels are limiting and achieve activation [16–18]. Previous studies showing uncompromised memory responses when CD28 co-stimulation was reduced have been interpreted as the result of increased TCR sensitivity and a redundancy for CD28 [4, 6, 10, 19]. However, these observations could also be the result of heightened sensitivity to co-stimulation providing more effective support for TCR signals even when CD28 ligand levels are low. Indeed, we previously found that blocking CD28 ligands with soluble CTLA4-Ig (Abatacept) impaired naïve responses in a dose-dependent manner but left memory responses largely intact. However, CRISPR knockout (KO) of CD28 ligands from the APC-ablated memory responses suggests that Abatacept treatment is an incomplete blockade and that memory T cells remain sensitive to the residual CD28 co-stimulation [20]. This observation is reminiscent of data from [6], where naïve responses were restricted to APCs that expressed high levels of CD28 ligand but memory T cells could respond to many APC subsets, including resting B cells and bone-marrow-derived macrophages, which express low levels of CD28 ligands.

Recently, anti-CTLA4 therapy has been used successfully to re-invigorate T cell-mediated anti-tumour immune responses by blocking CTLA4-mediated suppression of CD28 function. Here tumour infiltrating lymphocytes have an antigen-experienced memory phenotype [1, 21–27]. Moreover, CD28 signalling domains are also widely incorporated into the design of CAR T cells to enhance CAR T cell function in tumour immunotherapy [28–31]. Taken together, these observations allude to differences between naïve and memory T cells and highlight the need to better understand how varying the levels of TCR and CD28 stimulation affects T cell activation.

Here we have investigated the role of CD28 co-stimulation in human conventional CD4 + CD25− memory and naïve T cells by using different *in vitro* stimulation assays which vary the dose of TCR and CD28 stimuli alone and in combination. Our data reveal a clear impact of CD28 co-stimulation in driving memory T cell division and effector function, which differs from responses to TCR and by naïve cells. We observed that naïve T cells appeared to be more sensitive to TCR signals than memory T cells, whereas memory T cells could exploit CD28 engagement even in the absence of overt TCR signals. We also explored how the balance of TCR and CD28 signals influenced T cell responses and found that CD28 co-stimulation can synergize with, act independently of, and in some cases antagonize, aspects of the TCR stimulation. These data indicate an important role of CD28 co-stimulation for the control of specific memory T cell functions and suggest differences between naïve and memory T cells in the way they utilize TCR and CD28 signals.

## Methods

### Generating CD86-GFP and FcR cell lines

Full-length C-terminally GFP-tagged CD86 or human CD32 [Fcγ receptor II (FcR)] were cloned into the pMP71 retroviral vector to generate pMP71-CD86GFP or pMP71-FcR. Retroviral supernatants were obtained by transfection of Phoenix-Amphoteric packaging cells and used to transduce Chinese hamster ovary (CHO) cells (CHO-CD86GFP, CHO-FcR). For transduction, non-tissue culture-treated 24-well plates were coated with RetroNectin (TaKaRa) overnight at 4°C. 1 × 10^6^ CHO cells were added to 1 ml of retroviral supernatant per well and centrifuged at 2000 rpm, 32°C for 2 h. Twenty-four hours post-infection, the viral supernatant was removed and fresh media was added. Single-cell cultures of transduced CHO cells were produced by serial dilution and expanded to generate clonal lines.

DG75 KO lines (B cell lines with endogenous CD80 and CD86 knocked out) were generated by CRISPR-Cas9 targeting. sgRNAs were designed using Benchling containing a target sequence for CD80 (TTGAGGTATGGACACTTGGA) or CD86 (TTGACCTGCTCATCTATACA) and custom-made by Sigma-Aldrich. Cas9 (New England Biolabs) and sgRNA were mixed at a 1:3 molar ratio and incubated at 25°C for 30 min to form ribonucleoproteins. KO cell lines were generated using the Lonza Nucleofector 4D for nucleofection (program DS-104) with an SG Cell Line 4D-Nucleofector Kit (Lonza). Cells were allowed to recover for 3–5 days prior to screening and sorting for KO cells by flow cytometry. DG75 CD86GFP cell lines were generated by transducing DG75 B cells lines lacking CD80 and CD86 with CD86GFP retrovirus as indicated above.

### Tissue culture of cell lines

All cell lines were cultured at 37°C, 5% CO_2_, in a humidified atmosphere. CHO-K1 (ATCC, catalogue no. CCL-61) were cultured in Dulbecco’s modified Eagle’s medium (DMEM) supplemented with 10% Foetal calf serum (FCS) (Sigma-Aldrich), 2 mM L-glutamine, 100 U ml^−1^ penicillin and 100 mg ml^−1^ streptomycin all from Life Technologies (Gibco). Cells were routinely passaged at 1 in 10 via trypsinization. DG75 B cells (ATCC, catalogue no. CRL-2625) were cultured in Roswell Park Memorial Institute 1640 medium (RPMI) supplemented with 10% FCS (Lab Tech), 2 mM L-glutamine, 100 U ml^−1^ penicillin and 100 mg ml^−1^ streptomycin. Cells were split routinely at one in five with fresh complete media.

### Isolation of CD4± CD25− memory and naïve T cells

Total CD4 + CD25− T cells were derived from freshly isolated peripheral whole blood from healthy volunteers. Donors were 51% male with a mean age of 56 years and 49% female with a mean age of 56 years. Peripheral blood mononuclear cells (PBMCs) were extracted from whole blood via Ficoll gradient centrifugation (GE Healthcare) and T cells were isolated using immunomagnetic negative selection with a Custom Easy EasySep Human CD4 + CD25− T cell enrichment kit (STEMCELL). CD4 + CD25− memory and CD4 + CD25− naïve T cells were isolated from Leukocyte cones (NHS Blood and Transplant UK). PBMCs were isolated by Ficoll density gradient centrifugation. Memory T cells were isolated using EasySep Human Memory CD4 + T cell enrichment kit and naïve T cells were isolated using EasySep Human Naïve CD4 + T cell enrichment kit (STEMCELL). CD25 + memory and naïve T cells were then removed using the Custom Easy EasySep Human CD4 + CD25− T cell enrichment kit (STEMCELL).

### T cell stimulation assays

CHO or DG75 B cells were fixed in 0.025% glutaraldehyde (Sigma-Aldrich) for 3 min to preserve CD86 ligand or FcR expression for use as APCs in stimulation assays. Ratios of APC to T cells are indicated in figure legends. Soluble anti-CD3 (clone: OKT3, BioXCell), anti-CD28 (clone: 9.3, BioXCell) or TSST-1 (Toxin Technologies) were also used where indicated in figure legends at indicated concentrations. Where cells were stimulated with TSST-1, TCR Vβ2 + responding cells were gated for analysis. Prior to stimulation, CD4 + CD25− memory and naïve T cells were stained with Cell Trace Violet (CTV) for 20 min at 37°C. Assays were incubated at 37°C in 5% CO_2_ for indicated times, samples acquired a BD LSRFortessa II flow cytometer and analysed using FlowJo (TreeStar).

### Enumerating T cell division

Immediately prior to flow cytometry, a known number of AccuCheck Counting Beads (Thermo Fisher Scientific) was added to each sample. The absolute number of cells within the sample was calculated by expressing the number of cells identified as a fraction of the number of beads identified, multiplied by the number of beads added. This was also used to determine the absolute number of cells within each CTV peak.

## Results

### CD28 stimulation has greater impact on memory T cell commitment to division compared to naïve cells

**Figure 1.**
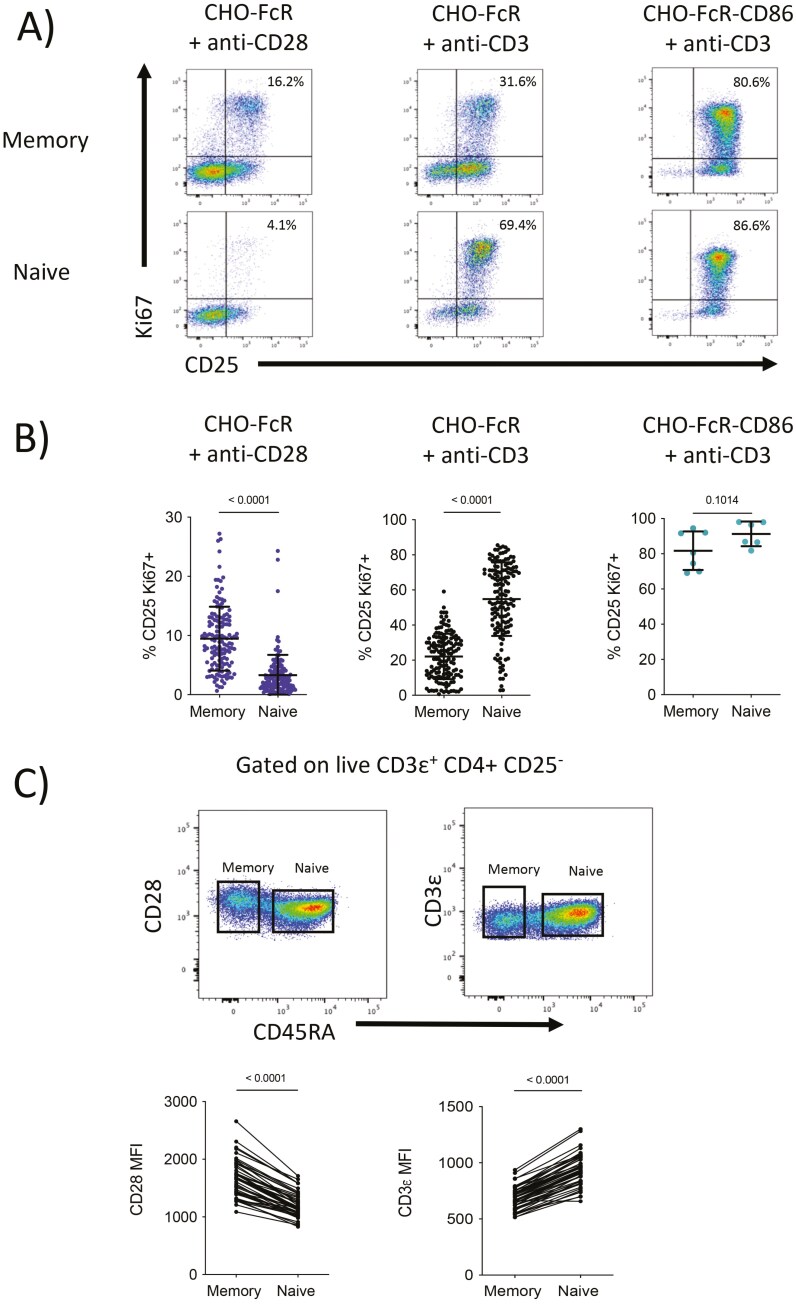
CD28 co-stimulation has a greater impact on memory CD4 + T cells compared to naïve. (A) Purified CD4 + CD25− T cells were co-stimulated 42 h with glutaraldehyde fixed, FcR transduced CHO cells, and 1 µg/ml soluble anti-CD3 (clone: OKT3) or 1 µg/ml soluble anti-CD28 (clone: 9.3) at a T:CHO ratio of 1:1.6. T cells were also stimulated with FcR and CD86-GFP co-transduced CHO cells and 1 µg/ml soluble anti-CD3. Analysis was performed by flow cytometry and responding cells were those co-expressing CD25 and Ki67. (B) Mean %CD25 + Ki67 + memory and naïve T cells are plotted ± SD (*n* = 6–141) and Mann–Whitney tests used to determine significance. (C) PBMC were analysed *ex vivo by* flow cytometry and median fluorescent intensity of surface CD28 and CD3ε compared between conventional memory and naïve T cells (*n* = 50–51) and paired *t*-tests used to determine significance.

To try and better understand the individual contribution of TCR and CD28 to purified CD25− memory and naïve commitment, we initially tested cross-linking of soluble anti-CD3 or anti-CD28 antibodies in the presence of CHO cells expressing Fc receptors. Whilst less commonly used, cross-linking CD28 alone is conceptually similar to CD3 cross-linking methods, which are widely employed. When cross-linked CD3 stimulation was used in the absence of co-stimulation we observed that naïve T cell responses clearly outperformed those from memory cells, generating a significantly higher percentage of cells committed to division as measured by CD25 + Ki67 + co-expression (**Fig. 1A**). In contrast, memory responses were stronger than naïve cells when CD28 was cross-linked in the absence of a TCR signal, albeit responses were lower in overall magnitude than those driven by the TCR (Fig. 1A). These results were repeated using a very large number of donors (**Fig. 1B**), showing the reproducibility of this finding. Control experiments where T cells were co-stimulated with anti-CD3 in the presence of CHO cells co-expressing both FcR and the CD28 ligand, CD86, showed robust and comparable commitment to division from both memory and naïve subsets. This latter observation highlights that different sensitivities to TCR and CD28 engagement by naïve and memory T cells can be obscured by traditional co-stimulation approaches where both signals are provided. *Ex vivo* phenotyping of PBMC also revealed consistently higher levels of CD28 in memory CD4 + T cells compared to their naïve counterparts in the same donor (**Fig. 1C**). Conversely, levels of CD3 were found to be higher on naïve T cells compared to memory. Together, these features may contribute to the different sensitivities observed in memory and naïve responses and suggest that surface CD28 is increased and CD3 decreased after the transition from naïve and memory. We also investigated ligation-induced downregulation of CD28 and CD3ε, however, similar downregulation patterns in both memory and naïve cells (**Supplementary Fig. S1A–C****)**.

Together these results suggested that despite similar responses from naïve and memory cells when both TCR and CD28 stimuli were present, memory T cells more effectively utilized CD28 co-stimulation to drive commitment to division whereas naïve cells responded more effectively to TCR stimulation.

### Division of memory T cells is greatest with strong CD28 co-stimulation and weaker TCR signals

**Figure 2.**
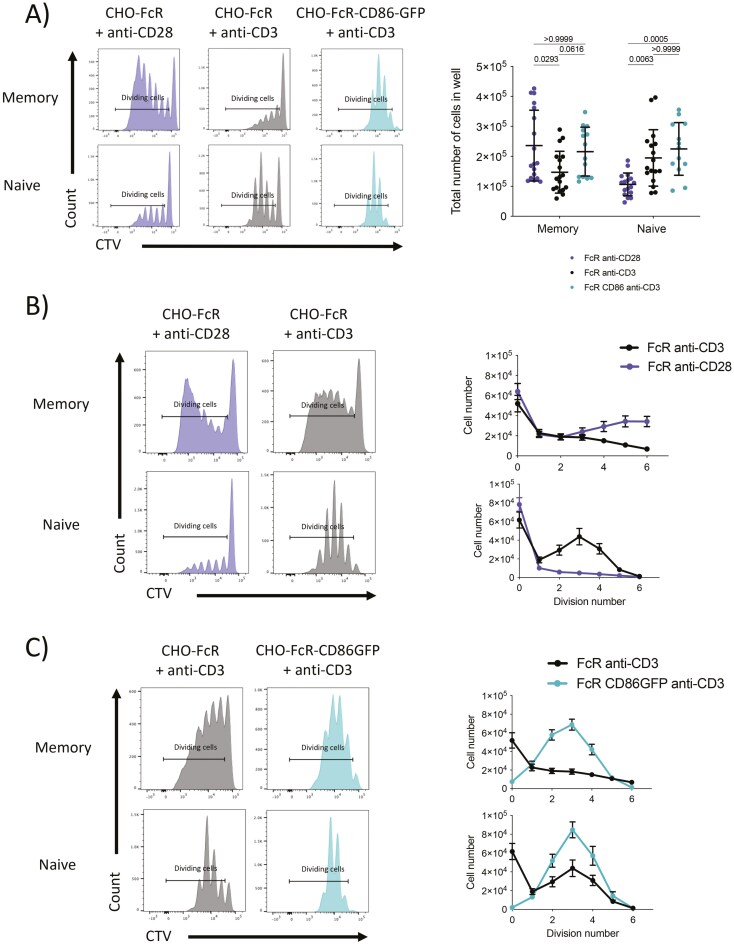
Division of memory T cells is greatest when challenged with strong CD28 co-stimulation and not strong TCR signals. Purified CD4 + CD25− memory and naïve T cells were labelled with CTV and stimulated with glutaraldehyde fixed, FcR transduced CHO cells, and 1 µg/ml soluble anti-CD3 (clone: OKT3) or 1 µg/ml anti-CD28 (clone: 9.3) at a T:CHO ratio of 1:1.6. Memory and naïve T cells were also stimulated with 1 µg/ml anti-CD3, with CHO cells transduced, and with FcR and CD86-GFP. Cells were cultured for 5 days before analysis by flow cytometry. Immediately before acquisition, samples were spiked with a known number of counting beads to enumerate (A) the total number of cells in the sample, (B) and (C) total number of cells per CTV peak. Graphs show mean ± SD (*n* = 13–18) and significance determined by the Kruskal–Wallis test.

The above experiments measured the initial commitment of T cells to divide as determined by CD25 + KI67 + cells, however, to follow the outcome of this commitment we measured both total cell accumulation and the distribution of cells per division after 5 days of stimulation using CTV dilution (gating shown in **Supplementary Fig. S2A**). This showed that for naïve T cells, the total number of responding cells was greatest when both TCR and CD28 signals were present (**Fig. 2A**). Interestingly, this was not significantly different when only cross-linked CD3 signals were present indicating that robust naïve responses can be generated using TCR alone (Fig. 2A). In contrast, significantly fewer naïve T cells responded after stimulation with CD28 alone suggesting that TCR signals are more important for the accumulation of large numbers of naïve T cells. In contrast, the total number of memory T cells was lowest in response to TCR cross-linking alone and significantly increased under conditions where a CD28 signal was present (Fig. 2A). Indeed, the number of memory cells generated after 5 days of cross-linking CD28 alone was comparable to conditions where both TCR and CD28 signals were present, (Fig. 2A) suggesting that memory cells could utilize CD28 to drive expansion of T cells. This was surprising since Fig. 1B showed cross-linking CD28 committed a smaller fraction of memory cells to divide when compared to strong TCR. However, as seen in the CTV plots (**Fig. 2B**), CD28 cross-linking resulted in an accumulation of memory cells in later divisions which was not seen in response to other stimuli. Therefore, whilst TCR alone committed more memory cells to division, CD28 supported greater expansion (Fig. 2B). Interestingly, the combination of CD3 and CD28 stimuli resulted in the most effective commitment to cell division but resulted in an intermediate number of cell divisions.

We performed further analysis of these experiments by plotting the total number of cells contained in each division, quantifying the accumulation of memory cells in later divisions in response to CD28 and comparing with TCR or in naïve T cell responses (Fig. 2B). We also compared cross-linked TCR stimulus with and without co-stimulation (**Fig. 2C**). This showed that co-stimulation enhanced the fraction of dividing memory T cells and altered the distribution of activated cells across CTV peaks. In contrast, the distribution of naïve cells per division was not influenced, albeit the number of cells in each division increased (Fig. 2C). These data supported the view that the provision of CD28 co-stimulation to naïve T cells increased proliferating cell numbers but did not enrich cells in later divisions.

We considered the possibility that resource competition, for example, for interleukin (IL)-2, might contribute to the limited expansion of memory T cells in response to both signals. We, therefore, compared intracellular staining of IL-2 in T cells in responding to CHO-FcR-CD86 + anti-CD3, FcR anti-CD3, FcR anti-CD28, and anti-CD3/CD28 dynabeads and the distribution of cells per division (**Supplementary Fig. S3A**  **and **B**)**. This suggested that IL-2 production was not directly correlated with the extent of cell division since FcR anti-CD28 and FcR anti-CD3 made similar levels of IL-2 per cell, yet accumulated differently. In addition, CD3/CD28 beads generated extensive proliferation yet made lower levels of IL-2 per cell. Nonetheless, this area requires further investigation of the kinetics of production and consumption to understand such influences in more detail.

Overall, the above data suggested that CD28 and TCR make different contributions to T cell responses in terms of commitment to division and subsequent proliferation and specifically that memory T cells appeared more able to effectively use CD28 to commit and expand activated cells.

### CD28 co-stimulation of memory T cells maintains cell survival and Ki67 expression

To determine why TCR-stimulated memory T cells did not accumulate in later divisions, we considered several possibilities: (i) that cells may have reduced ability to sustain cell division, (ii) cells may be subject to increased cell death in later divisions, or (iii) cells may experience asynchronous commitment with different stimuli and continue to enter division thereby affecting the overall division profile.

**Figure 3.**
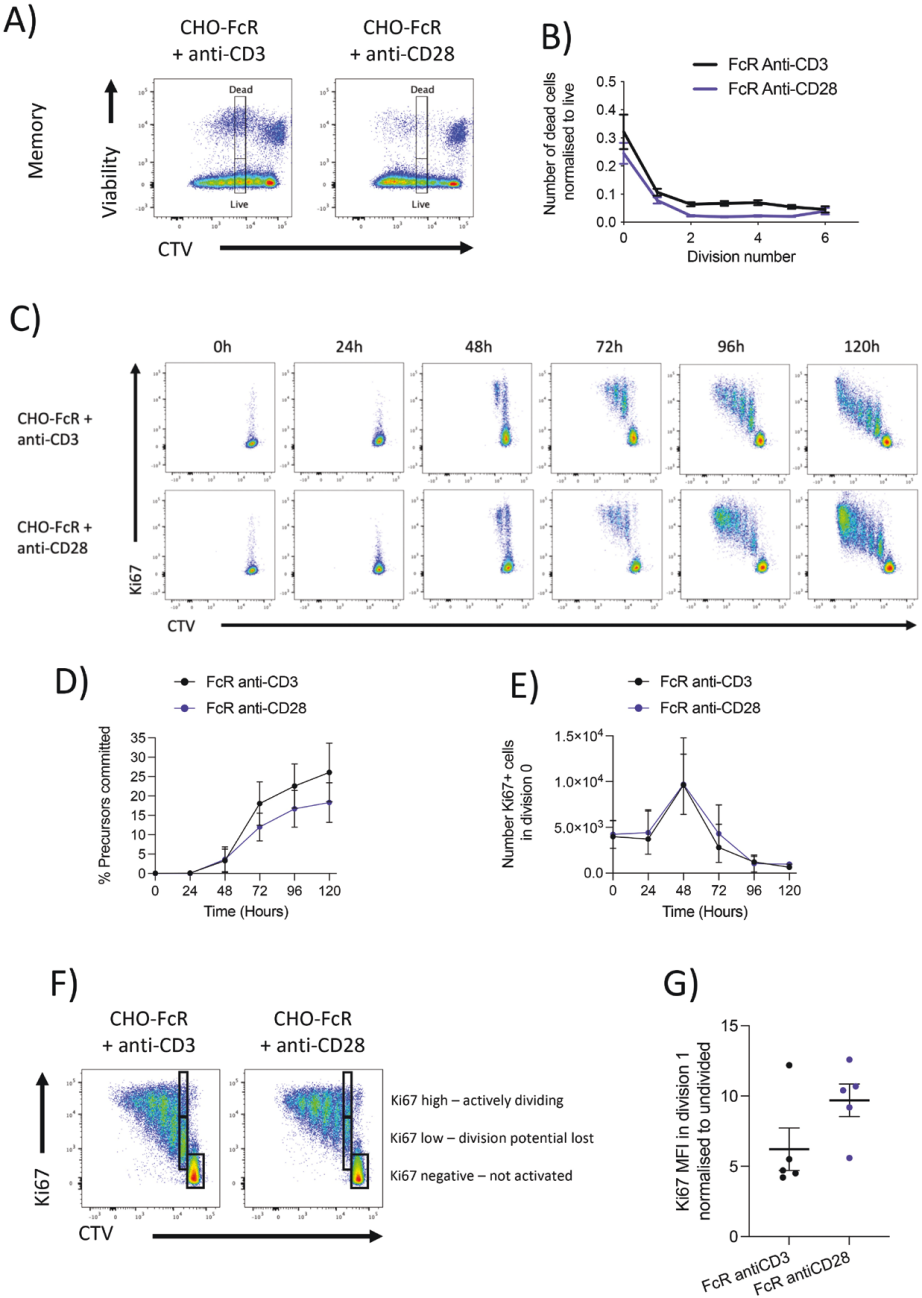
CD28 co-stimulation maintains division potential of memory T cells. Purified CD4 + CD25− memory T cells were labelled with CTV and stimulated with glutaraldehyde fixed, FcR transduced CHO cells, and 1 µg/ml soluble anti-CD3 (clone: OKT3) or 1 µg/ml anti-CD28 (clone: 9.3) at a T:CHO ratio of 1:1.6. Cells were cultured for 5 days or at indicated timepoints before analysis by flow cytometry. Immediately before acquisition, samples were spiked with a known number of counting beads to enumerate cell numbers. (A) Representative live/dead staining on proliferating cells, (B) number of dead cells normalized to total number of cells per division, (C) time course of CTV and Ki67 staining, (D) cumulative percentage of precursors that committed to making at least 1 division at indicated timepoints, (E) number of undivided cells expressing Ki67 at indicated timepoints, (F) representative Ki67 staining, and (G) Ki67 MIF of cells in division 1 normalized to Ki67− cells in division 0. Graphs show mean ± SD (*n* = 3–18).

To investigate whether TCR-stimulated memory T cells underwent increased cell death, we stimulated memory T cells with CHO-FcR and either soluble anti-CD3 or anti-CD28 for 5 days. We calculated the total number of cells within each division and stratified live from dead cells with a viability dye (**Fig. 3A**). We then determined the fraction of dead cells in each division and plotted it as a live:dead ratio. This showed that TCR-stimulated cells had a modestly increased frequency of cell death in all divisions compared to CD28-stimulated cells (**Fig. 3B**), however, it is unlikely that this was sufficient to explain the lack of memory T cells in later divisions in response to TCR.

To address the question of asynchronous commitment, we performed a kinetic analysis of memory T cells stimulated with either anti-CD3 or anti-CD28 and sampled division over 5 days (**Fig. 3C**). We calculated the total number of precursor cells that had contributed to division at different timepoints and expressed this as a percentage of the total number of precursor cells (**Fig. 3D**). As expected, the total commitment was somewhat greater in response to TCR signals, however, the rate of memory T cell commitment did not differ between TCR and CD28 and very few cells appeared to commit beyond 72 h of stimulation for either stimulus. This was also confirmed by counting the number of Ki67 + cells in division ‘0’ over time as a measure of cells that had recently become triggered but not yet divided (**Fig. 3E**). Thus, cross-linked CD3 and CD28 show a similar number of Ki67 + in division ‘0’ over time with a synchronous peak at 48 h and few Ki67 + undivided cells appearing post-72 h. This suggests that T cells committed to division within the first 72 h of stimulation with no evidence for asynchronous commitment between the two stimuli.

We also analysed the Ki67 expression level since it is thought that Ki67 expression is maintained in cells that are actively dividing and is downregulated as division potential is lost. Therefore, Ki67 levels may indicate cells’ potential to divide. Since Ki67 has a half-life of approximately 3 days, analysis levels of Ki67 in division ‘1’ after 5 days of stimulation can be used to determine if committed cells had lost division potential during the response. As seen in **Fig. 3F and G**, Ki67 levels in division ‘1’ were lower under TCR stimulation alone compared with CD28 stimulation alone. This raises the possibility that TCR-stimulated memory cells readily lose the ability to divide, whereas CD28-stimulated cells are more likely to make subsequent divisions.

Together these data suggest that the ability of memory T cells to accumulate in later divisions is likely the result of a combination of factors including enhanced potential to divide with CD28 signals (Fig. 3F) alongside a slightly enhanced survival profile in each division.

### Balance between TCR and CD28 signals affects number of divisions made by memory T cells but not naïve

**Figure 4.**
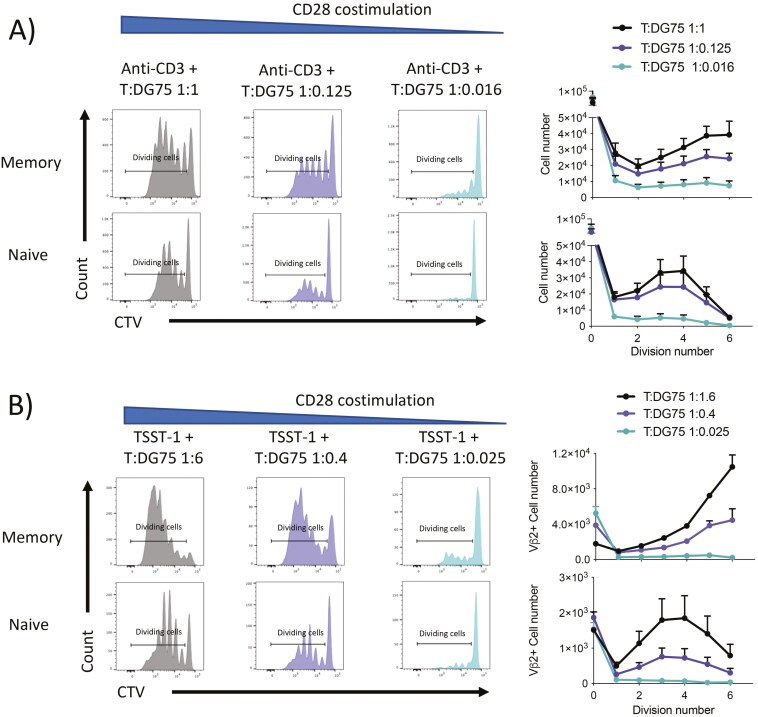
Number of divisions made by memory T cells is sensitive to the dose of CD28. Purified CD4 + CD25− memory or naïve T cells were labelled with CTV and stimulated for 5 days with a titration of glutaraldehyde-fixed CD86-GFP transduced DG75 B cells and (A) 0.1 µg/ml soluble anti-CD3 (clone: OKT3) or (B) 0.5 pg/ml of TSST-1. Where TSST-1 was used, Vβ2 + T cells were gated. Immediately before acquisition, samples were spiked with a known number of counting beads to enumerate total number of cells per CTV peak. Graphs show mean ± SD. *n* = 3–7.

The above data provided a framework for considering the functions of CD28 and TCR using a CHO cell-based model. However, we also tested these ideas using B cells as a more physiological APC type. We hypothesized that altering the balance between TCR and CD28 signals should impact T cell division of memory and naïve in line with the above findings. We, therefore, used a fixed dose of soluble anti-CD3 and varied co-stimulation using human DG75 B cells transduced with the CD86-GFP-tagged ligand to tune CD28 co-stimulation. We observed more memory T cells accumulated in later divisions at higher levels of CD28 co-stimulation, whereas, the distribution of activated naïve T cells remained broadly unchanged (**Fig. 4A**). These observations were also recapitulated by substituting soluble anti-CD3 for the MHC-dependent superantigen, TSST-1 (**Fig. 4B**, gating shown in **Supplementary Fig. S2****B**), demonstrating that these features are maintained in both MHC-dependent and non-MHC-dependent responses. Thus, these data indicate that accumulation in later divisions is characteristic of memory cells responding to CD28 co-stimulation. In contrast, increasing CD28 co-stimulation in naïve T cells has increased the number of activated cells but did not result in the extended division seen in memory T cells.

**Figure 5.**
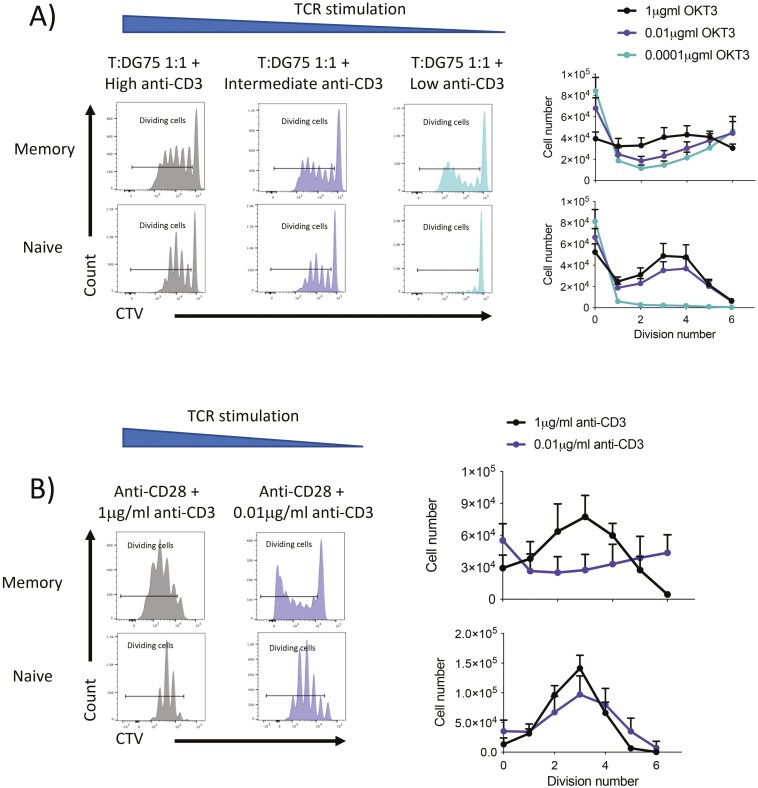
**Number of divisions made by memory T cells is sensitive to the dose of CD28 relative to TCR.** Purified CD4 + CD25− memory or naïve T cells were labelled with CTV and stimulated for 5 days with (A) glutaraldehyde fixed, CD86-GFP transduced DG75 B cells, and a titration of soluble anti-CD3 at a T cell:DG75 ratio of 1:1 or (B) with FcR transduced glutaraldehyde-fixed CHO and cells 1 µg/ml soluble anti-CD28 (clone: 9.3) and a titration of soluble anti-CD3. Immediately before acquisition, samples were spiked with a known number of counting beads to enumerate total number of cells per CTV peak. Graphs show mean ± SD (*n* = 3–7).

We also tested conditions where TCR signals were favoured by increasing the dose of soluble anti-CD3 while fixing levels of CD86 co-stimulation. As expected, increasing the dose of TCR stimulus increased the number of activated cells. However, the division profile of activated naïve T cells was not influenced by increasing TCR dose (**Fig. 5A**). In contrast, activated memory cells preferentially accumulated in later divisions when TCR signals were low, e.g. where CD28 signals were high relative to TCR. Notably, when higher doses of TCR were supplied, e.g. where CD28 signals are low relative to TCR, the extent of division was attenuated suggesting that the balance between TCR and CD28 signals may ultimately control the division profile (Fig. 5A). This impact of changing the balance between TCR and CD28 stimulation on the extent of cell division was also seen using the CHO-FcR system where a fixed dose of anti-CD28 and a titration of anti-CD3 gave similar results (**Fig. 5B**). Together, these data suggest that the division profile of activated memory T cells can be tuned by the relative quantity of CD28 compared to TCR, where TCR signals antagonize the extent of division, whereas the division profile of naïve T cells does not change with the strength of either signal.

### CD28 and TCR stimulation induces distinct T cell phenotypes in memory T cells

**Figure 6.**
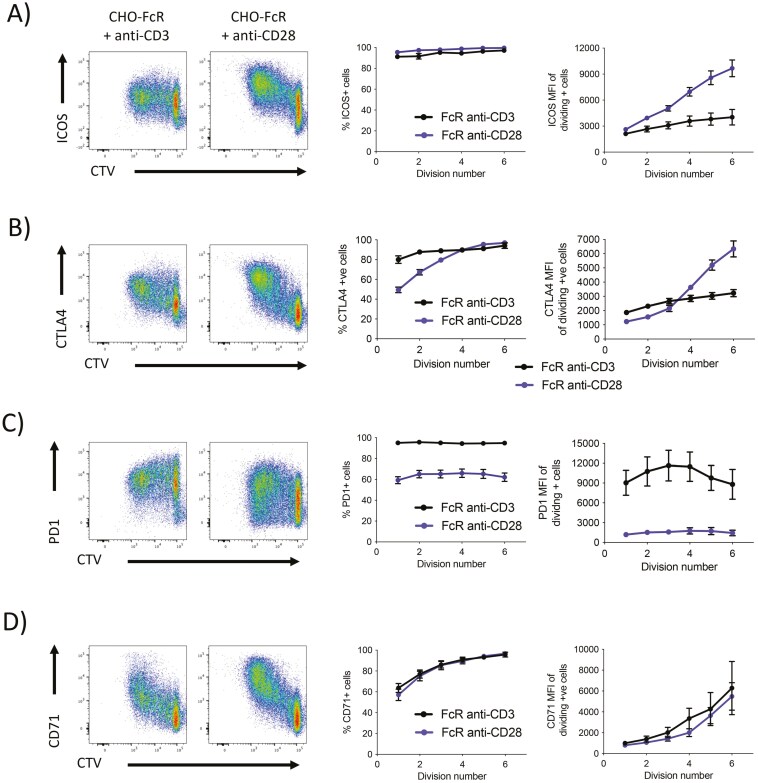
CD28 and TCR differentially control expression patterns of effector markers in memory T cells. Purified CD4 + CD25− memory T cells were labelled with CTV and stimulated with glutaraldehyde-fixed FcR transduced CHO cells and 1 µg/ml soluble anti-CD3 (clone: OKT3) or 1 µg/ml anti-CD28 (clone: 9.3) at a T:CHO ratio of 1:1.6. Cells were cultured for 5 days before analysis by flow cytometry. Graphs show mean percentage positive cells per division and mean MFI of positive cells per division ± SD (*n* = 8).

We also compared the relative contribution of TCR and CD28 stimulation on T cell phenotype and cytokine production. Since these changes were not a major feature of naïve cells (**Supplementary Fig. S4**) we focused on memory responses to TCR and CD28 engagement. We observed that cross-linked CD28 signals induced greater expression levels of ICOS and CTLA4, compared to TCR, a difference which became progressively more pronounced as cells divided (**Fig. 6A and B**). In contrast, expression levels and induction of PD1 were favoured by TCR signals (**Fig. 6C**). Additionally, CD71 (transferrin receptor) showed similar patterns of expression with both TCR or CD28 activation (**Fig. 6D**).

**Figure 7.**
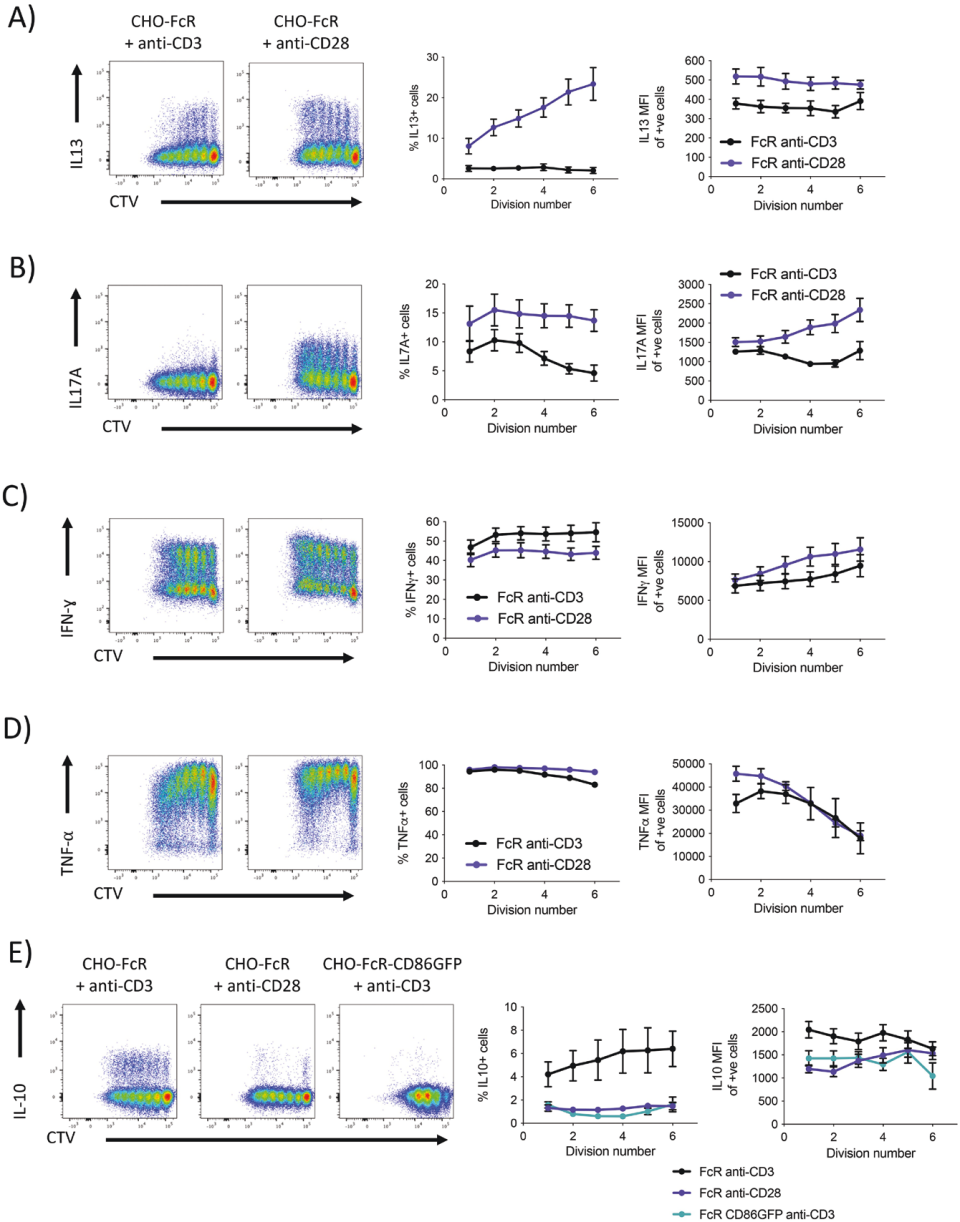
CD28 and TCR differentially control expression patterns of effector cytokines in memory T cells. Purified CD4 + CD25− memory T cells were labelled with CTV and stimulated with glutaraldehyde-fixed FcR transduced CHO cells and 1 µg/ml soluble anti-CD3 (clone: OKT3) or 1 µg/ml anti-CD28 (clone: 9.3) or FcR-CD86GFP transduced CHO cells and 1 µg/ml soluble anti-CD3 at a T:CHO ratio of 1:1.6. Cells were cultured for 5 days and 4 h before analysis by flow cytometry, cells were treated with BFA, PMA, and ionomycin. Graphs show mean percentage positive cells per division and mean MFI of positive cells per division ± SE (*n* = 4–8).

We also measured cytokine production, observing that CD28 co-stimulation significantly contributed to IL-13 and IL-17 expression and was capable of inducing more robust cytokine expression compared to TCR stimulation (**Fig. 7A and B**). It was also notable that CD28 induced a division-linked increase in the frequency of IL-13-expressing cells but a division-linked increase in the level of IL-17A expression. Similar to CD71, TNF-α and IFN-γ were not obviously favoured by either signal (**Fig. 7C and D**), whereas, similar to PD1, IL-10 expression was clearly favoured by TCR signals (**Fig. 7E**) and largely absent from CD28 stimulation. However, the addition of CD28 co-stimulation in the presence of cross-linked CD3 also inhibited IL-10 expression suggesting that CD28 stimulation can antagonize some aspects of TCR-driven functions. Taken together our data show that TCR and CD28 stimulation can have different effects on T cell proliferation, cytokine production, and phenotype.

## Discussion

The CD28 pathway is an important therapeutic target which can be manipulated to increase or decrease its function. Therapeutic targeting of ligands by CTLA4-Ig fusion proteins (Abatacept) or blocking CTLA4 with antibodies (e.g. Ipilimumab) have been used in autoimmunity and cancer to inhibit or invigorate CD28 signals, respectively. Nonetheless, how CD28 contributes to outcomes of T cell stimulation is still not fully understood, and the impact of CD28 co-stimulation often generates conflicting data, for example, regarding roles in Treg or IL-17 induction [32–41]. One central issue is distinguishing between CD28-related functions and those of the TCR in typical settings where both pathways are in play and therefore understanding the contribution of each signal. This concept is important because both of these stimuli are naturally highly variable. TCR affinity and levels of peptide–MHC complexes vary widely, similarly, CD28 has two different affinity ligands CD80 and CD80 whose patterns and level of expression vary both in terms of cell type and induction by inflammatory signals. Thus, T cells continually experience a variable balance between TCR and CD28, yet our understanding of their respective impact is poor.

Here, by combining a series of *in vitro* stimulation assays, we have attempted to dissect the relative contributions of TCR and CD28. Our data suggest that the division of labour of CD28 and TCR is not equivalent between memory and naïve subsets and that CD28 is more influential for the control of effector cytokine production and extended division of activated memory cells which can be fine-tuned by the dose of CD28 relative to TCR. In contrast, inhibitory/regulatory pathways such as IL-10 production and PD1 expression were more robustly observed following TCR signals and in the case of IL-10 apparently opposed by CD28.

Somewhat surprisingly our data indicate that naïve T cells may be more responsive to TCR signals, whereas memory cells are more responsive to CD28 stimulation. It is notable that these differences are not obvious in true co-stimulation experiments, because when both signals are provided it becomes difficult to determine the contribution of each pathway. However, across a large number of donors, we observed that naïve T cells were capable of responding more effectively to TCR alone compared to memory cells, at least in terms of commitment to division as indicated by CD25 and Ki67 expression. The use of such early markers of activation is potentially useful because unlike later outcomes of activation, such as overall T cell proliferation, these early indicators are less affected by cell survival or cell division rate. Thus, these data show early fundamental differences between TCR and CD28 use in naïve and memory T cells.

We observed that cross-linking CD3 initially committed more memory T cells to division at 48 h, compared to cross-linking CD28. However, the total number of memory cells at 5 days was actually greater in response to CD28 and counting the number of cells within each CTV division peak showed an accumulation of memory cells in later divisions in response to CD28 cross-linking that was not seen in naïve cells or in response to TCR alone, suggesting CD28 sustains division in memory T cells. While the mechanism for this observation is uncertain, we observed small gains in viability and in the maintenance of Ki67 levels via CD28, which would promote accumulation in later divisions. This is consistent with *in vivo* studies, where blockade of the CD28 ligands in murine antigen-specific memory responses reduced the extent of cell division in activated cells [42]. Extended division was also evident in CTLA4^−/−^ CAR T cells, which showed increased population doublings upon *in vitro* stimulation [43], again implying CD28 can control the extent of division.

Despite our use of *in vitro* reductionist systems to assess T cell responses, our observations were consistent across several different systems. Accordingly, we recapitulated our findings from CHO cell systems with B cells as APC using an MHC-dependent superantigen as well as with anti-CD3. By varying the relative levels of TCR and CD28 signals our data suggest it is not simply the quantity of co-stimulation but the balance between TCR and CD28 signals which is an important determinant of responses. Supporting this idea, Agarwal et al. [43], showed deletion of PD1, a negative regulator of TCR, attenuated the proliferative benefit afforded by *CTLA4* deletion in CAR T cells, where CTLA4 and PD1 indirectly regulate TCR and CD28 signals. Another recent study also reported increased division profiles of CAR T cells when fusing copies of the CTLA4 cytoplasmic domain to the CAR receptor [44]. This reduced surface expression of the CAR is likely due to internalization and degradation via the CTLA4 domains [2, 45, 46] and is therefore consistent with the possibility that changing the level of ‘TCR-like’ CAR signalling, affected the balance between a TCR and CD28 signals.

Finally, we also observed that cytokine production and T cell phenotype were differentially controlled by TCR and CD28. In general, CD28 promoted higher levels of expression of several markers, supporting its role in T cell effector functions. Importantly, many markers reached maximum expression during later divisions exclusively in response to co-stimulation. This highlights the significance of CD28-driven repeat divisions for inducing effective effector responses. This was again consistent with observations from [43] where greater expression of IL-13 and ICOS was observed in *CTLA4*^−*/*−^ CAR. Importantly, not all markers were favoured by CD28. CD71, IFN-γ, and TNF-α were not differentially expressed in response to either signal, and PD1 and IL-10 were favoured by TCR signals. This highlights both the shared and non-overlapping functions of CD28 and TCR and surprisingly, in the case of IL-10 expression, antagonism by the provision of CD28 signals.

TCR and CD28 co-stimulation forms a critical axis of regulating T cell responses and is the molecular basis for the classical ‘two signal model’. Nonetheless, the amount of signal delivered by the TCR and CD28 is highly variable and context-dependent with the independent contributions of these pathways still poorly understood. We have used a variety of model systems to probe these outcomes and our data indicate differences between naïve and memory T cells in their sensitivity to these signals and how the balance between CD28 and TCR signalling affects both proliferative potential and T cell phenotype. These fundamental concepts may be significant in a number of areas of immunology including cancer immunotherapy or immunosuppressive treatments. For example, the increased sensitivity of memory T cells to CD28 stimulation suggests they may be more effectively targeted by CD28-directed interventions such as ligand blockade, CD28 antagonists or enhancing CTLA4 function. Conversely, naïve T cells may be more sensitive to TCR-targeted interventions such as calcineurin inhibitors, or potentially peptide–MHC modulation. Ultimately, a better understanding of how the balance between TCR and CD28 signals influences immune outcomes offers further possibilities for targeted immune regulation.

## Supplementary Material

kyaf006_suppl_Supplementary_Figure_1

kyaf006_suppl_Supplementary_Figure_2

kyaf006_suppl_Supplementary_Figure_3

kyaf006_suppl_Supplementary_Figure_4

kyaf006_suppl_Supplementary_Fig_Legends

## Data Availability

The data underlying this article will be shared upon reasonable request to the corresponding author.
