# Supplementary figures and images for: CD28 and TCR differentially impact naïve and memory T cell responses
Source: Discov Immunol. 2025 Apr 22;4(1):kyaf006. doi: 10.1093/discim/kyaf006 (PMC12150779; doi:10.1093/discim/kyaf006)

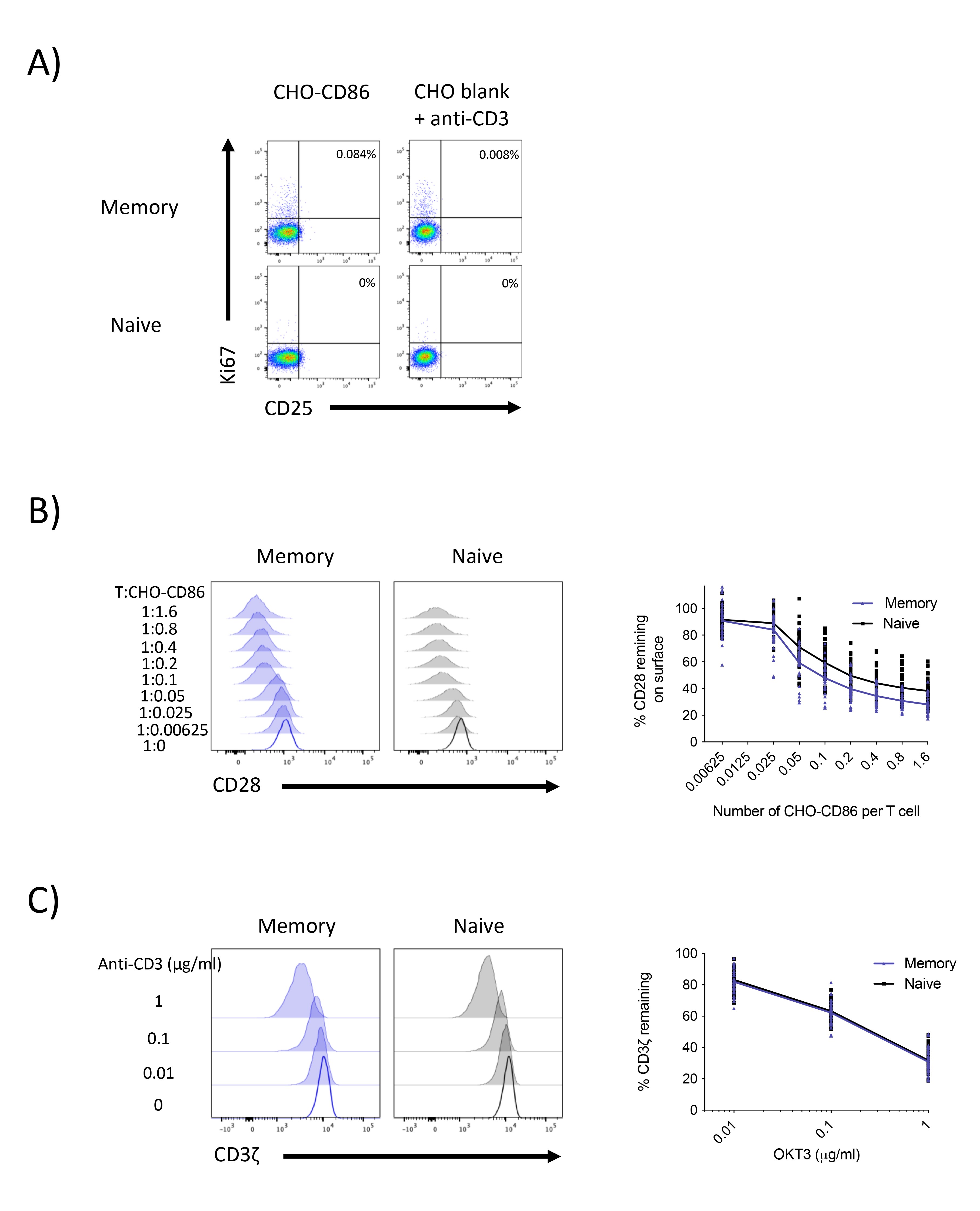

Supplement: kyaf006_suppl_Supplementary_Figure_1 [file kyaf006_suppl_supplementary_figure_1.jpeg]

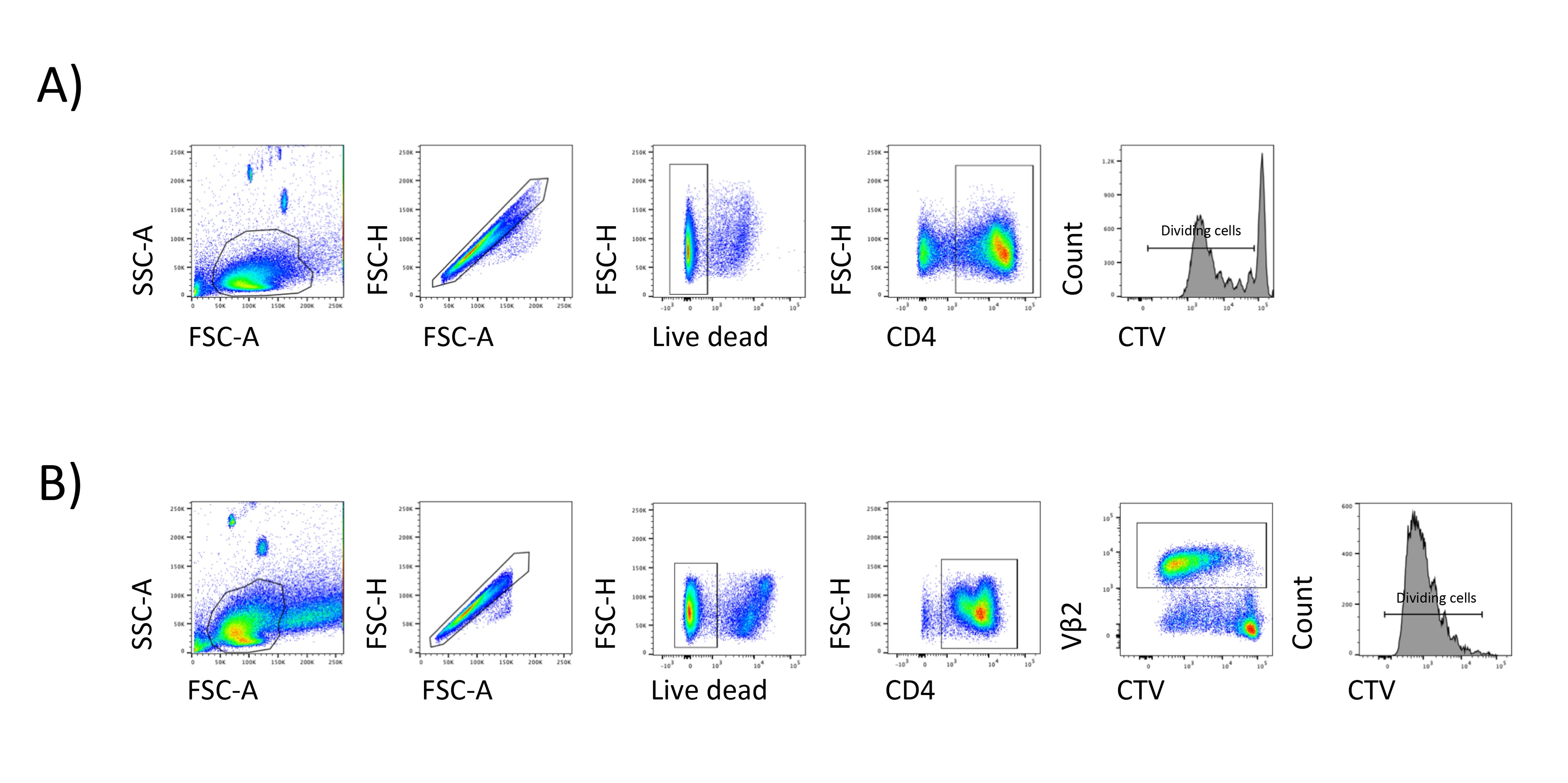

Supplement: kyaf006_suppl_Supplementary_Figure_2 [file kyaf006_suppl_supplementary_figure_2.jpeg]

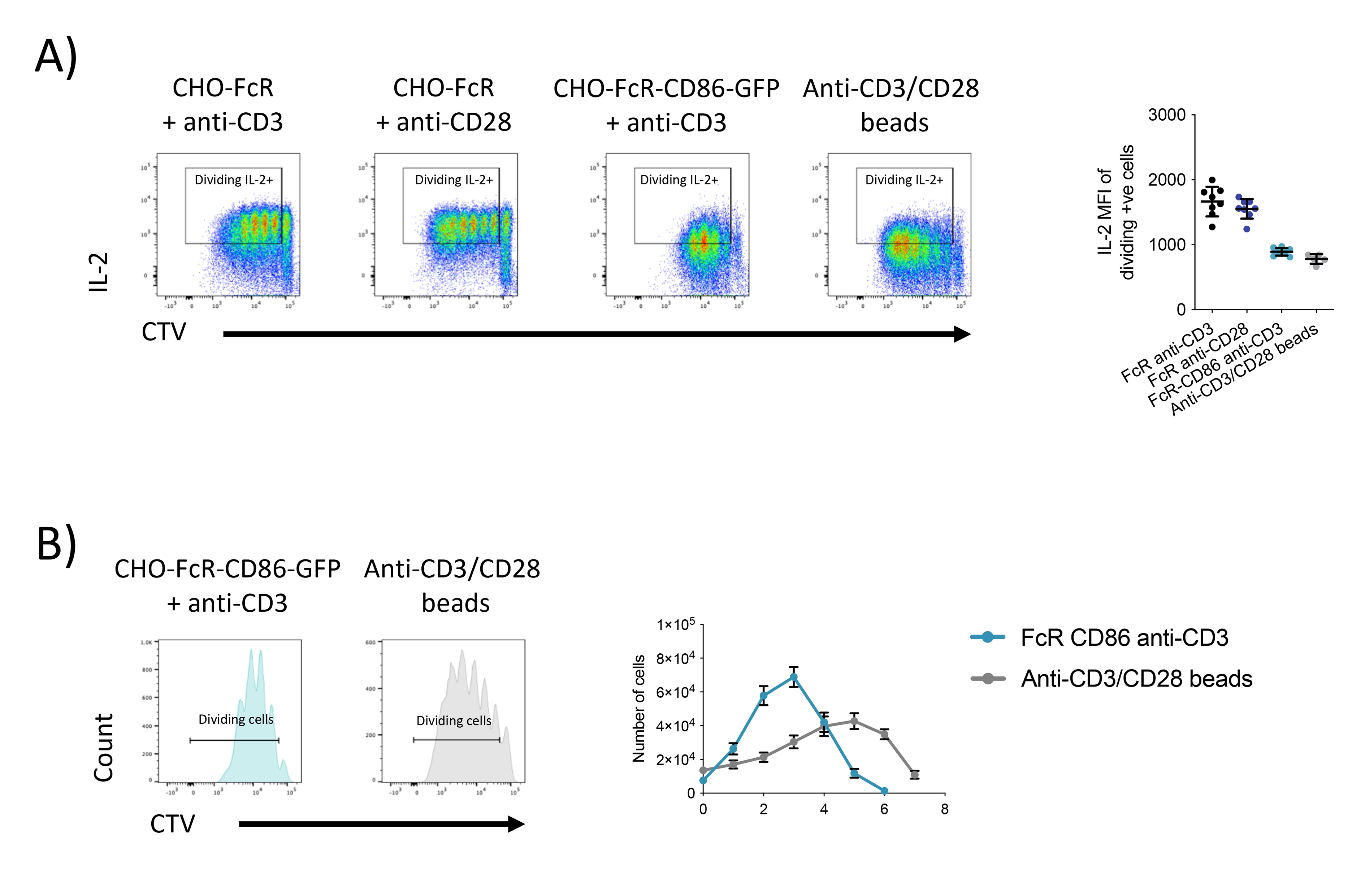

Supplement: kyaf006_suppl_Supplementary_Figure_3 [file kyaf006_suppl_supplementary_figure_3.jpeg]

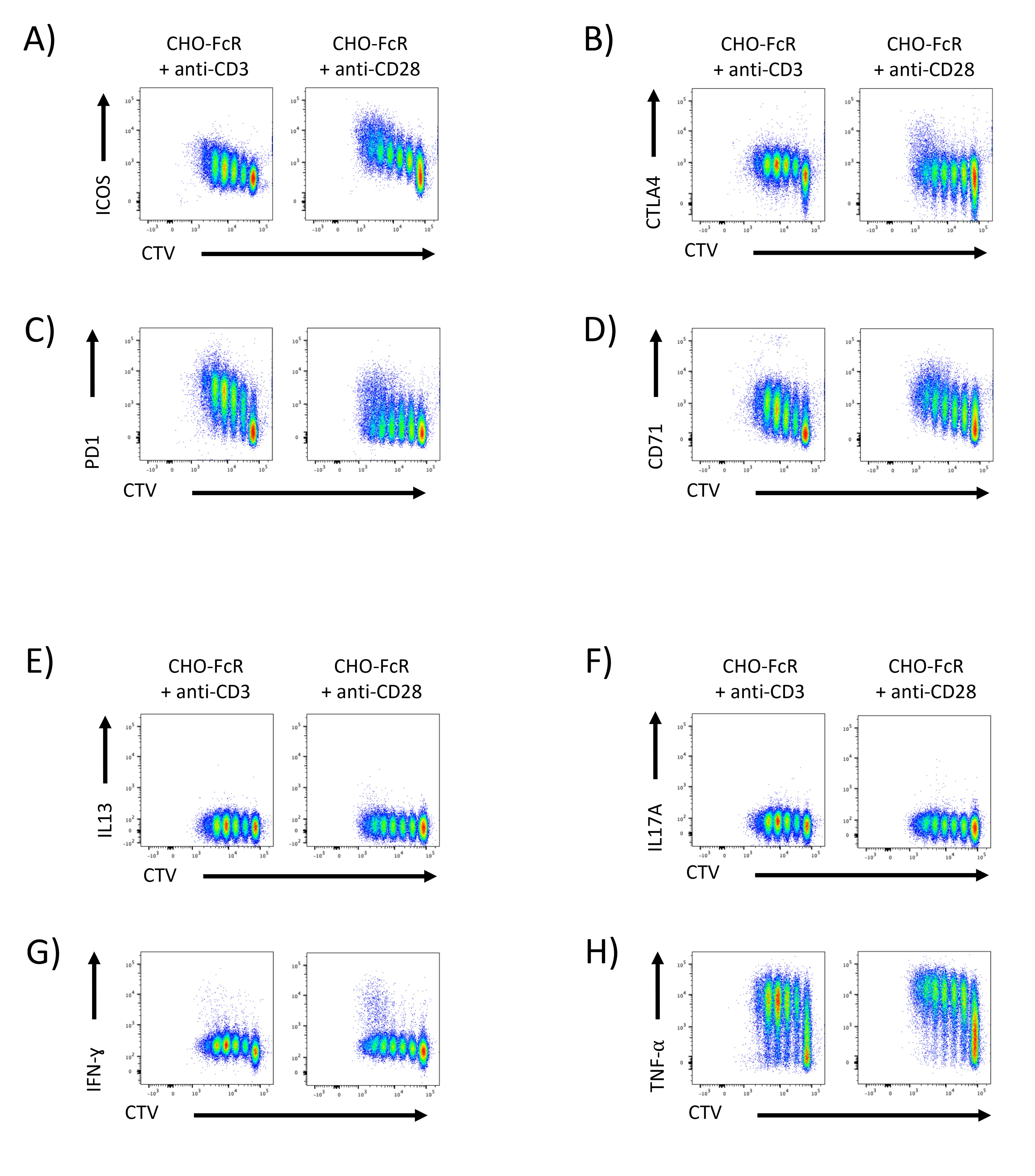

Supplement: kyaf006_suppl_Supplementary_Figure_4 [file kyaf006_suppl_supplementary_figure_4.jpeg]
