# Supplementary material for: CD28 and TCR differentially impact naïve and memory T cell responses
Source: Discov Immunol. 2025 Apr 22;4(1):kyaf006. doi: 10.1093/discim/kyaf006 (PMC12150779; doi:10.1093/discim/kyaf006)
Supplement: kyaf006_suppl_Supplementary_Fig_Legends [file kyaf006_suppl_supplementary_fig_legends.docx]

**Supplementary figure 1. Ligation-induced CD28 and CD3ζ downregulation is comparable between Memory and Naïve T cells.** CD4+ CD25- T cells were cultured for 42h in the presence of glutaraldehyde fixed CD86 transduced CHO cells or soluble anti-CD3 (Clone:OKT3) before analysis by flow cytometry. A) Representative FACS plots showing limited background activation of Memory or Naïve T cells. B) Surface levels of CD28 on CD25- Ki67- Memory and Naïve T cells following culture with a titration of CHO-CD86. C) Intracellular levels of CD3ζ CD25- Ki67- Memory and Naïve T cells following culture with a titration anti-CD3. Graphs show mean % receptor remining relative to untreated controls n=36-37.

**Supplementary figure 2. Representative gating hierarchy for experiments involving CTV dilution.** CTV labelled CD4+ CD25- Memory or Naïve T cells were cultured separately for 5 days under conditions stated in relevant figures before analysis by flow cytometry. A) Representative FACS plots showing identification of dividing T cells. B) Representative FACS plots showing identification of dividing Vβ2+ T cells.

**Supplementary figure 3. Expansion of activated T cells is not directly correlated with IL-2 production.** Purified CD4+ CD25- Memory T cells were labelled with CTV and stimulated with glutaraldehyde fixed FcR transduced CHO cells and 1µg/ml soluble anti-CD3 (Clone: OKT3) or 1µg/ml anti-CD28 (Clone: 9.3) or FcR-CD86GFP transduced CHO cells and 1µg/ml soluble anti-CD3 at T:CHO ratios of 1:1.6 or with anti-CD3/CD28 dynabeads at a T:bead ratio of 1:0.25. A) Cells were cultured for 5 days and 4 hours before analysis by flow cytometry, cells were treated with BFA, PMA and ionomycin. Graph show mean percentage positive cells +/- SD n=5-8. B) Immediately before acquisition, samples were spiked with a known number of counting beads to enumerate total number of cells per CTV peak. Graphs show mean +/- SD n=11-14.

**Supplementary figure 4. Naïve T cells express low levels of effector markers and cytokines.** A-D) Purified CD4+ CD25- Naive T cells were labelled with CTV and stimulated with glutaraldehyde fixed FcR transduced CHO cells and 1µg/ml soluble anti-CD3 (Clone: OKT3) or 1µg/ml anti-CD28 (Clone: 9.3) at a T:CHO ratio of 1:1.6. Cells were cultured for 5 days before analysis by flow cytometry. E-H) As above, except 4 hours before analysis by flow cytometry, cells were treated with BFA, PMA and ionomycin.
